# ASO Author Reflections: Effectiveness of SPECT/CT Imaging for Sentinel Node Biopsy Staging of Primary Cutaneous Melanoma and Patient Outcomes

**DOI:** 10.1245/s10434-021-11026-6

**Published:** 2021-11-29

**Authors:** Marc Moncrieff, Sarah Pywell, Andrew Snelling, Matthew Gray, David Newman, Clare Beadsmoore, Martin Heaton, Davina Pawaroo

**Affiliations:** 1 Department of Plastic & Reconstructive Surgery, Norfolk and Norwich University Hospitals NHS Foundation Trust, Norwich, UK; 2 Norwich Medical School, University of East Anglia, Norwich, UK; 3 Department of Nuclear Medicine, Norfolk and Norwich University Hospitals NHS Foundation, Norwich, UK

## Past

Since its development, sentinel node biopsy (SNB) has evolved to become the standard of care for primary cutaneous melanoma and is incorporated in the current AJCC classification system.^1^ Accurate preoperative lymphoscintigraphy (LSG) is essential for successfully performing SNB.^2^ The introduction of coregistered single-photon emission computed tomography with integrated computed tomography (SPECT/CT) has greatly improved the accuracy of localisation of the sentinel lymph node(s) (SLN) for primary cutaneous melanoma. Whereas the improvement in accuracy is generally acknowledged, surgeons were becoming increasingly aware that more lymph nodes were being identified preoperatively and sent for analysis, which was impacting the productivity of their respective pathology services. It is our practice to counsel patients preoperatively in the clinic that occasionally multiple lymph nodes or multiple basins would be identified and that a conversation before surgery after the scan would be necessary in this scenario. As a consequence, a few patients would change their mind and decide not to proceed with the planned SNB on the day of surgery after consultation with their surgeon due to the perceived risk of increased postoperative morbidity and long-term quality of life issues. Examples of this situation would be a thin pT1b primary in a young, fit patient with either drainage to multiple neck and/or parotid nodes or a thin primary draining to both the groin and pelvis. For the group that decides to cancel their SNB, there are concerns that the patient is missing their important staging procedure, with additional concerns regarding timely access to adjuvant systemic therapy and overall prognosis.

## Present

We undertook a retrospective study of prospectively collected data to investigate the effectiveness of SPECT/CT imaging for SNB staging of primary cutaneous melanoma based on a large cohort (n = 1552) treated at an academic, tertiary referral cancer centre during a 10-year period.^3^ The key findings were that 95% (1446/1522) of patients underwent a successful SNB procedure. Significantly more sentinel nodes were identified by the SPECT/CT protocol (3 vs. 2; *p* < 0.0001). More patients were cancelled in the SPECT/CT cohort (9.3% vs. 2.5%; *p* < 0.0001). Head & neck, lower limb, and AJCC IB primaries were significantly less likely to proceed to SNB. SPECT/CT identified significantly more positive SNBs (20.9% vs. 16.5%; *p* = 0.038). SPECT/CT imaging was associated with improved disease-free (hazard ratio [HR] = 0.74; 95% confidence interval [CI]: 0.54–1.0; *p* = 0.048) and disease-specific survival (HR = 0.48; 95% CI: 0.3–0.78; *p* = 0.003). Patients who did not proceed to SNB had a significantly increased nodal relapse rate (23.5% vs. 6.8%; HR = 3.4; 95% CI: 1.9–6.2; *p* < 0.0001) compared with those who underwent SNB.

## Future

It is well documented that the original sentinel node biopsy technique has/had a definite false-negative rate. Indeed, many original detractors of the technique made a great deal out of this very issue. The final report of the MSLT-1 study showed that the patients who had a false-negative SNB (i.e., developed a nodal recurrence after an initial negative sentinel node biopsy) had a much worse prognosis than either the sentinel node positive patients, or those who did not have sentinel node biopsy but developed a recurrence (Morton et al. 2014, p. 606; Figs. 3c–d, cohort 4).^4^ Other data from the MSLT-1 study have shown that the head and neck region is particularly prone to inaccuracy.^5^ Data from Sydney also has shown that approximately one-third of nodal recurrences in previous false-negative sentinel nodes are due to lack of accuracy of the LSG.^6^

We suggest that preoperative SPECT/CT imaging of sentinel node(s) for melanoma patients represents a major improvement in accuracy in terms of the anatomical detail that is obtained. Our data have shown that this increased anatomical detail results in more sentinel nodes and more nodal basins being identified. Logic dictates that the result would be an increased positive sentinel node detection rate, and our data confirm this. We have shown that this translates into an improved disease-free and melanoma-specific survival and, whilst this is not an RCT, we would strongly suggest that this improved survival benefit results from the earlier harvest of the nodal metastases for a small, but important subgroup of patients.

With regards to the reasons for cancellation of the sentinel node biopsy on the day of surgery, our data suggest that the overriding reason for cancelling the procedure is that the number of nodes identified by the scan is deemed excessive by both the patients and their surgeons, and this is a result of the increased anatomical detail seen in the SPECT/CT scan. The data also shows that the following subgroups of patients are more likely to decide not to proceed with a SNB: younger; lower limb or head and neck primaries; thinner, lower risk primaries; multiple nodes and/or basins. Our data also have shown is that this increased anatomical detail comes at a potential cost, in that more patients are deciding not to proceed with their surgery, and as a result, some of those patients are presenting with nodal metastases, which are more likely to otherwise have been identified if they had proceeded with the SNB.

Ultimately, we believe that the addition of SPECT/CT to our preoperative SLN localisation protocol is a progressive step. However, the improved accuracy that it affords comes with an increased workload for pathology departments and an increased risk of cancellation of the SNB procedure on the day of surgery, which in turn has a negative impact on nodal relapse-free survival. These data would suggest evaluating the true effectiveness of SPECT/CT imaging in SNB staging of melanoma is complex that would be better explained by a formal health economics analysis. The data also highlight that accurate, noninvasive biomarkers for staging primary cutaneous melanoma patients are required.
